# Surge in Ceftriaxone-Resistant *Neisseria gonorrhoeae* FC428-Like Strains, Asia-Pacific Region, 2015−2022

**DOI:** 10.3201/eid3008.240139

**Published:** 2024-08

**Authors:** Leshan Xiu, Lulu Zhang, Junping Peng

**Affiliations:** School of Global Health, Shanghai Jiao Tong University School of Medicine, Shanghai, China (L. Xiu);; National Institute of Pathogen Biology, Chinese Academy of Medical Sciences & Peking Union Medical College, Beijing, China (L. Zhang, J. Peng)

**Keywords:** gonorrhea, ceftriaxone-resistant, *Neisseria gonorrhoeae*, FC428-like strain, Asia-Pacific Region, antimicrobial resistance, China

## Abstract

Ceftriaxone-resistant *Neisseria gonorrhoeae* FC428-like strains have disseminated across the Asia-Pacific region, with a continuous rise in prevalence during 2015–2022. To mitigate the effect of these strains, we advocate for enhanced molecular diagnostics, expanded surveillance networks, and a regionally coordinated effort to combat the global spread of FC428-like strains.

*Neisseria gonorrhoeae* infections represent an urgent public health threat, compounded by the alarming surge in strains resistant to ceftriaxone, the last line of defense for gonorrhea treatment (*1*). Before 2015, ceftriaxone resistance and treatment failures were sporadically reported globally. However, since 2015, the ceftriaxone-resistant FC428 clone carrying the mosaic *penA*-60.001 allele, initially identified in Japan (*2*), has spread nationally and internationally. Given this context, the World Health Organization’s global surveillance of gonococcal antimicrobial resistance (AMR) needs to urgently expand internationally to provide essential data for developing effective management guidelines and public health policies (*3*). 

The Asia-Pacific region, housing two thirds of the world’s population and 10 of the least developed countries (*4*), is recognized as a regional hotspot for the emergence and spread of AMR. Indeed, recent evidence indicates that AMR in gonococci typically originates in an area the World Health Organization has deemed the Western Pacific Region before subsequently spreading internationally (*5*). Understanding the prevalence and dissemination of FC428-like strains in the Asia-Pacific region is therefore crucial for studying their origins and implementing measures to curtail their ongoing global spread. Our study offers a comprehensive analysis of the global spread of the ceftriaxone-resistant FC428-like strains, with a focus on prevalence, genetic diversity, and geographic distribution.

## The Study

**Figure 1:**
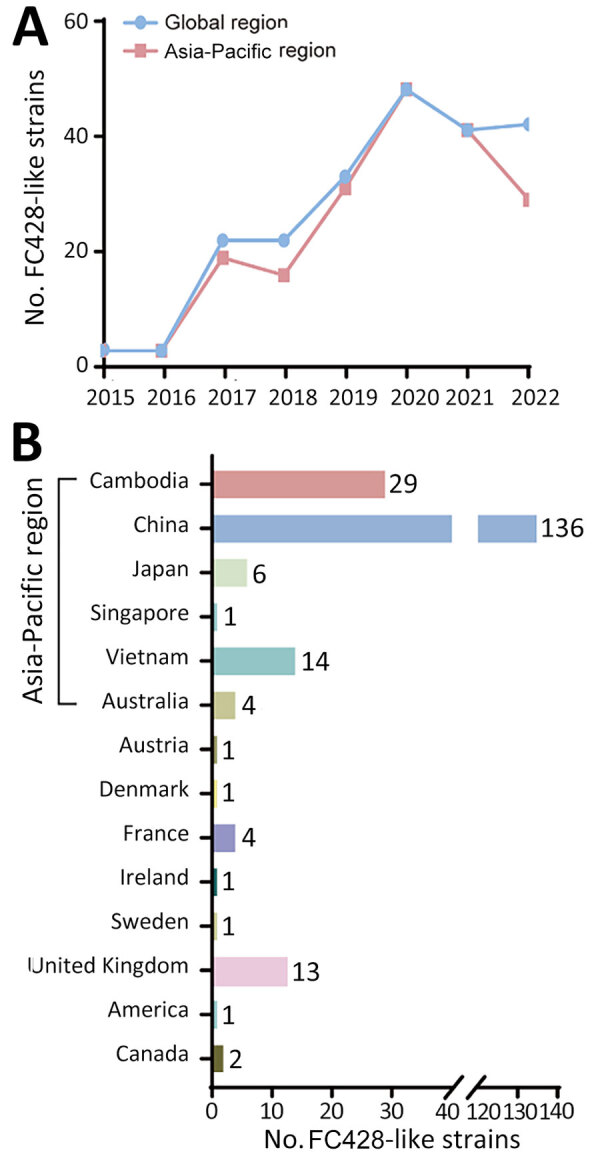
Global dissemination of ceftriaxone-resistant *N. gonorrhoeae* FC428-like strains. A) Trends in the prevalence of FC428-like strains from global and Asia-Pacific regions during 2015–2022. B) Number of FC428-like strains identified across 14 countries.

We acquired a sample library of FC428-like strains, incorporating data from our previously published effort (*6*), publicly available worldwide information collected over an 8-year period (*7*), and previously unpublished data from a comprehensive surveillance program in China conducted during 2019–2021 (National Center for Biotechnology Information Sequence Read Archive accession no. PRJNA560592). We identified 214 FC428-like strains across 14 countries (Appendix Table, Figure), most from Asia (186 strains), followed by Europe (21 strains), Oceania (4 strains), and North America (3 strains), suggesting circulation in the Asia-Pacific region (Figure 1). The prevalence of FC428-like strains has continuously increased during 2015–2022 (Figure 1, panel A), possibly because of an actual increase in cases but also potentially because of advancements in sequencing technology and increased use of more straightforward AMR tests. In a surveillance initiative for FC428-like strains, researchers determined minimal inhibitory concentrations for ceftriaxone (*8*). In another study, researchers categorized isolates as ceftriaxone susceptible or ceftriaxone resistant according to the latest clinical breakpoints (version 14.0) from the European Committee on Antimicrobial Susceptibility Testing (*9*). Based on those breakpoints, ceftriaxone resistance is defined as a MIC of ≥0.25 mg/L. Antimicrobial susceptibility analysis on 210 strains revealed that 195 (92.86%) displayed a ceftriaxone-resistant phenotype, and 15 strains exhibited susceptibility. Caution is warranted when interpreting the clinical relevance of ceftriaxone resistance with *penA*-60.001. We classified 211 FC428-like strains (3 strains lacking sequence information) into 23 multilocus sequence typing sequence types (STs) (Appendix Table). ST1903 was predominant (44.08%, 93/211), followed by ST13871 (10.90%, 23/211), ST1600 (7.11%, 15/211), ST7365 (6.64%, 14/211), ST8123 (5.69%, 12/211), and ST7363 (5.21%, 11/211).

**Figure 2:**
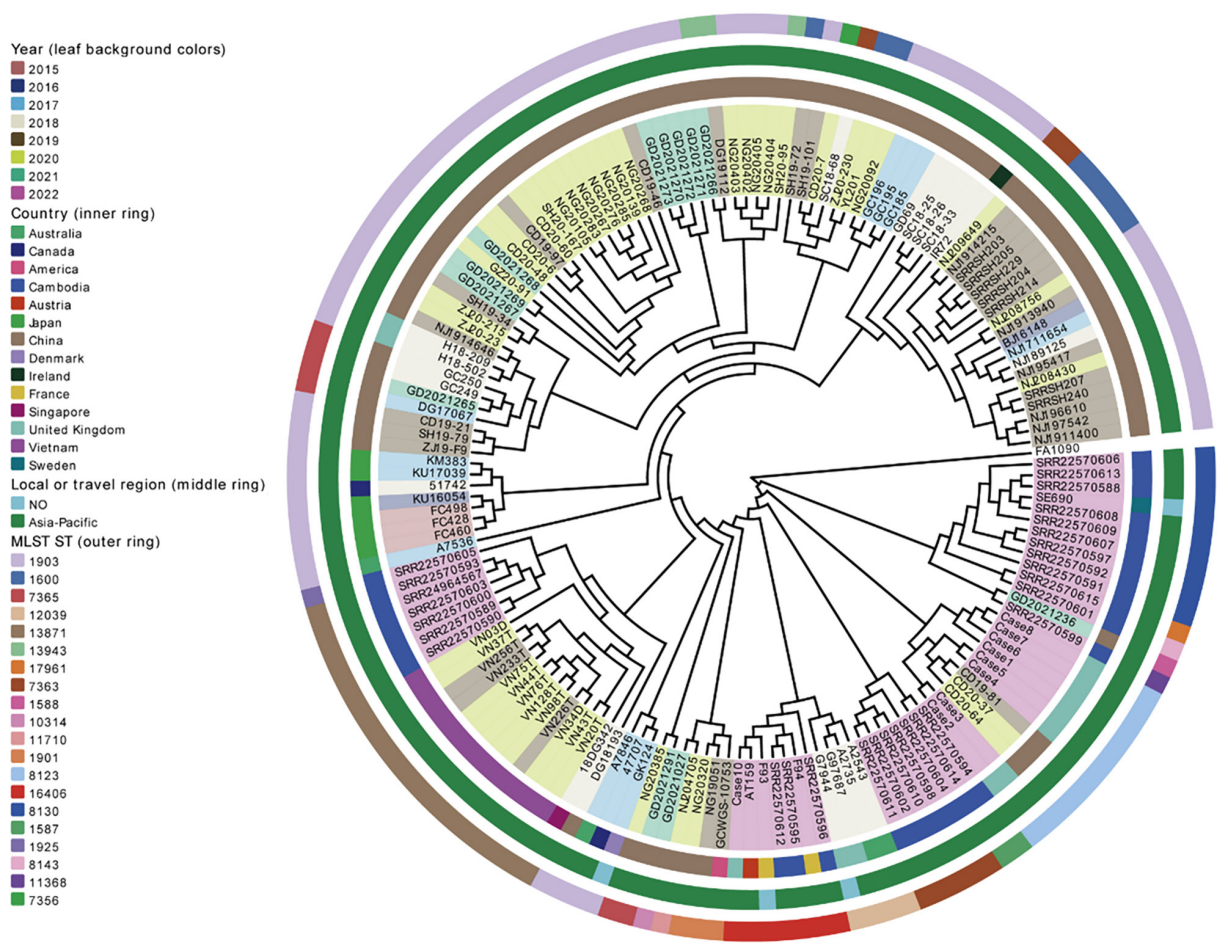
Data from study of the surge in ceftriaxone-resistant *Neisseria gonorrhoeae* FC428-like strains in the Asia-Pacific region, 2015−2022. Phylogenetic analysis of globally disseminated gonococcal FC428-like strains. MLST, multilocus sequence typing; ST, sequence type.

We obtained whole-genome sequence data from either our previously unpublished dataset or those we downloaded from public databases (Appendix Table). We extracted total genomic DNA from each bacterial isolate by using the QIAamp DNA Mini Kit (QIAGEN, https://www.sigmaaldrich.com) and prepared libraries for sequencing by using the Nextera XT DNA Library Preparation Kit (Illumina, https://www.illumina.com). We used the Illumina NovaSeq 6000 platform to execute sequencing. As of November 23, 2023, we could identify only 158 (73.83%) of the 214 FC428-like strains on the basis of available whole-genome sequencing data. The Asia-Pacific region hosted the most FC428-like strain genomes (86.08%, 136/158). We linked the remaining 22 strains, isolated from outside the Asia-Pacific region, to infections associated with travel or contact history in that region, suggesting a potential epidemiologic link. We generated a concatenate superset of single-nucleotide polymorphisms to measure genetic variations, following previously described methods (*6*). We then constructed a maximum-likelihood phylogenetic tree based on genomewide single-nucleotide polymorphisms by using PhyML 3.0. We conducted a whole-genome phylogenetic analysis, categorizing 158 strains into 2 major lineages and 10 distinct clades (Figure 2). Lineage A accounted for 51.9% (82/158), comprising clade 1, primarily isolated in Japan, and clade 2, mainly composed of FC428-like strains isolated in China. Lineage B consisted of 8 clades isolated from 12 different countries, but primarily from Cambodia and Vietnam. FC428-like strains from Europe, Oceania, and North America appeared interspersed in the lineage B phylogeny, suggesting multiple introductions of FC428-like strains into those countries.

Strains from different countries exhibited diverse STs, suggesting a common ancestry for these FC428-like strains that subsequently disseminated and evolved in other areas. China and Cambodia displayed the richest variety of sequence types. The diversity within the FC428-like population mirrors the global population, indicating potential multiple sources for lineages found within regional collections. Traditional sequence types have evolved further into FC428-like strains, particularly those susceptible to ceftriaxone, through the acquisition of *penA*-60.001 and homologous recombination of the core genome, resulting in the emergence of new clones with increased resistance. Those events lead to development of new clones at the global level, followed by the erosion of signals of clonality through recombination and, in some identifiable cases, the formation of new clonal clusters.

To mitigate the effect of newly emerged and future clones, additional research is required to understand the evolutionary mechanisms involved in the emergence of the new *N. gonorrhoeae* lineages that contribute to global dissemination over relatively short timeframes. Large-scale genomic data provide valuable insights into the identification and characterization of FC428-like strains, enhancing our understanding of their global distribution and associated epidemiologic links. The strains forming lineage B phylogeny are sourced primarily from Cambodia and Vietnam, again highlighting distinct epidemiologic links (*10*,*11*). This reinforcing evidence strengthens the argument for the interconnectedness of FC428-like strains across different regions and underscores the significance of international travel and contact in their dissemination.

## Conclusions

Effective disease control measures are crucial for addressing *N. gonorrhoeae* infections, given the substantial increase in identifying persons with ceftriaxone-resistant FC428-like strains observed globally over the past decade (*12*). This study provides insights into the prevalence, genetic diversity, and geographic distribution of these strains, contributing to a collective understanding of the current state of AMR in *N. gonorrhoeae* and the strategies needed to address this pressing public health issue.

Ceftriaxone resistance mediated by the *penA*-60.001 allele has been increasing, emphasizing the need for closer attention to identifying and surveilling of FC428-like strains. How, then, do we tackle the threat of ceftriaxone-resistant FC428-like strains? First, in addition to continuing to use antimicrobial susceptibility testing methods to detect novel resistance mechanisms, we recommend expanding access to molecular diagnostics (*13*) to provide routine ceftriaxone-resistant testing as a minimum for all persons investigated for gonorrhea. Tailoring regimens on the basis of individual resistance profiles could maximize therapeutic efficacy while minimizing the risk of contributing to further resistance. Second, governments in the Asia-Pacific region should build on existing networks and local public health laboratories to expand capacity for ongoing surveillance and contribute data to the global fight against AMR (*3*). Deploying genomic AMR surveillance as a leapfrog technology (*1*,*14*), skipping over the targeted-molecular expansion of isolate-based phenotypic surveillance, can differentiate between strains within closely related clades dominating in specific countries, enabling detailed transmission mapping surpassing limitations of standard typing methods. Third, creating an enabling environment supported by appropriate resourcing is essential to fostering multisectoral partnerships to drive innovative solutions delivered through responsive and well-resourced health services. Finally, a regionally coordinated effort, driven by clear targets and sustainability, and built on a framework that facilitates communication and governance, will strengthen the fight against the global dissemination of ceftriaxone-resistant *N. gonorrhoeae* FC428-like strains.

Appendix Additional information for surge in ceftriaxone-resistant *Neisseria gonorrhoeae* FC428-like strains, Asia-Pacific region, 2015−2022.

## References

[1] Sánchez-Busó L, Yeats CA, Taylor B, Goater RJ, Underwood A, Abudahab K, et al. A community-driven resource for genomic epidemiology and antimicrobial resistance prediction of *Neisseria gonorrhoeae* at Pathogenwatch. Genome Med. 2021;13:61. 10.1186/s13073-021-00858-2 33875000 PMC8054416

[2] Lee K, Nakayama SI, Osawa K, Yoshida H, Arakawa S, Furubayashi KI, et al. Clonal expansion and spread of the ceftriaxone-resistant *Neisseria gonorrhoeae* strain FC428, identified in Japan in 2015, and closely related isolates. J Antimicrob Chemother. 2019;74:1812–9. 10.1093/jac/dkz129 31002306

[3] Unemo M, Lahra MM, Escher M, Eremin S, Cole MJ, Galarza P, et al. WHO global antimicrobial resistance surveillance for *Neisseria gonorrhoeae* 2017-18: a retrospective observational study. Lancet Microbe. 2021;2:e627–36. 10.1016/S2666-5247(21)00171-3 35544082

[4] Yam ELY, Hsu LY, Yap EP, Yeo TW, Lee V, Schlundt J, et al. Antimicrobial Resistance in the Asia Pacific region: a meeting report. Antimicrob Resist Infect Control. 2019;8:202. 10.1186/s13756-019-0654-8 31890158 PMC6921568

[5] Unemo M, Bradshaw CS, Hocking JS, de Vries HJC, Francis SC, Mabey D, et al. Sexually transmitted infections: challenges ahead. Lancet Infect Dis. 2017;17:e235–79. 10.1016/S1473-3099(17)30310-9 28701272

[6] Yuan Q, Li Y, Xiu L, Zhang C, Fu Y, Jiang C, et al. Identification of multidrug-resistant *Neisseria gonorrhoeae* isolates with combined resistance to both ceftriaxone and azithromycin, China, 2017-2018. Emerg Microbes Infect. 2019;8:1546–9. 10.1080/22221751.2019.1681242 31661379 PMC6830194

[7] Ouk V, Pham CD, Wi T, van Hal SJ, Lahra MM; EGASP Cambodia working group. The Enhanced Gonococcal Surveillance Programme, Cambodia. Lancet Infect Dis. 2023;23:e332–3. 10.1016/S1473-3099(23)00479-6 37549683

[8] Xiu L, Wang L, Li Y, Hu L, Huang J, Yong G, et al. Multicentre clinical evaluation of a molecular diagnostic assay to identify *Neisseria gonorrhoeae* infection and detect antimicrobial resistance. Int J Antimicrob Agents. 2023;61:106785. 10.1016/j.ijantimicag.2023.106785 36918087

[9] The European Committee on Antimicrobial Susceptibility Testing. Breakpoint tables for interpretation of MICs and zone diameters. Version 14. 2024 [cited 2024 Jan 1] https://www.eucast.org/clinical_breakpoints

[10] Day M, Pitt R, Mody N, Saunders J, Rai R, Nori A, et al. Detection of 10 cases of ceftriaxone-resistant *Neisseria gonorrhoeae* in the United Kingdom, December 2021 to June 2022. Euro Surveill. 2022;27:2200803. 10.2807/1560-7917.ES.2022.27.46.2200803 36398578 PMC9673238

[11] Maubaret C, Caméléna F, Mrimèche M, Braille A, Liberge M, Mainardis M, et al. Two cases of extensively drug-resistant (XDR) *Neisseria gonorrhoeae* infection combining ceftriaxone-resistance and high-level azithromycin resistance, France, November 2022 and May 2023. Euro Surveill. 2023;28:2300456. 10.2807/1560-7917.ES.2023.28.37.2300456 37707979 PMC10687985

[12] Lahra MM, Ryder N, Whiley DM. A new multidrug-resistant strain of *Neisseria gonorrhoeae* in Australia. N Engl J Med. 2014;371:1850–1. 10.1056/NEJMc1408109 25372111

[13] Tickner JA, Lahra MM, Whiley DM. The need for a commercial test using the penA60 allele to identify ceftriaxone-resistant *Neisseria gonorrhoeae.* Lancet Infect Dis. 2022;22:1271–2. 10.1016/S1473-3099(22)00520-5 35961361

[14] Golparian D, Unemo M. Now Is the Time to Implement Whole Genome Sequencing in the Global Antimicrobial Resistance Surveillance for *Neisseria gonorrhoeae?* EClinicalMedicine. 2019;7:11–2. 10.1016/j.eclinm.2019.02.002 31193652 PMC6539334

