# Supplementary material for: Surge in Ceftriaxone-Resistant Neisseria gonorrhoeae FC428-Like Strains, Asia-Pacific Region, 2015−2022
Source: Emerg Infect Dis. 2024 Aug;30(8):1683–6. doi: 10.3201/eid3008.240139 (PMC11286042; doi:10.3201/eid3008.240139)
Supplement: Appendix — Additional information for surge in ceftriaxone-resistant Neisseria gonorrhoeae FC428-like strains, Asia-Pacific region, 2015−2022. [file 24-0139-Techapp-s1.pdf]

Article DOI: http://doi.org/10.3201/eid3008.240139

EID cannot ensure accessibility for supplementary materials supplied by authors. Readers who have difficulty accessing supplementary content should contact the authors for assistance.

Surge in Ceftriaxone-Resistant *Neisseria gonorrhoeae* FC428-Like Strains, Asia-Pacific Region, 2015−2022

Appendix

Appendix Table. Global characteristics of FC428-like strains carrying the mosaic *penA*-60.001 allele from a study of ceftriaxone-resistant *Neisseria gonorrhoeae* FC428-like strains surge in the Asia-Pacific region

Number	Isolate	Country	Year	Local, travel or contact region	CRO (mg/L)	MLST	*pen*A allele	Genomic accession no.	References
1	FC428	Japan	2015	Asia-Pacific	0.5	1903	60.001	SRR6238586	(*1*)
2	FC460	Japan	2015	Asia-Pacific	0.5	1903	60.001	SRR6238585	(*1*)
3	FC498	Japan	2015	Asia-Pacific	0.75	1903	60.001	DRR099844	(*1*)
4	KU16054	Japan	2016	Asia-Pacific	0.5	1903	60.001	DRR099846	(*1*)
5	BJ16148	China	2016	Asia-Pacific	0.5	1903	60.001	GCA_014700555.1	(*2*)
6	SZ20	China	2016	Asia-Pacific	0.5	1903	60.001	NA	(*3*)
7	SRRSH180	China	2017	Asia-Pacific	0.5	1903	60.001	NA	(*3*)
8	BJ17003	China	2017	Asia-Pacific	0.5	1903	60.001	NA	(*4*)
9	BJ17031	China	2017	Asia-Pacific	0.5	1903	60.001	NA	(*4*)
10	BJ17038	China	2017	Asia-Pacific	0.5	1903	60.001	NA	(*4*)
11	BJ17033	China	2017	Asia-Pacific	1	1903	60.001	NA	(*4*)

Number	Isolate	Country	Year	Local, travel or contact region	CRO (mg/L)	MLST	*pen*A allele	Genomic accession no.	References
12	BJ17037	China	2017	Asia-Pacific	0.5	1903	60.001	NA	(*4*)
13	MM08	China	2017	Asia-Pacific	0.25	1903	60.001	SRR16847908	(*5*)
14	SH 40	China	2017	Asia-Pacific	≥1	NA	60.001	NA	(*6*)
15	SH 41	China	2017	Asia-Pacific	≥1	NA	60.001	NA	(*6*)
16	SH 48	China	2017	Asia-Pacific	≥1	NA	60.001	NA	(*6*)
17	F90	France	2017	Asia-Pacific	0.5	1903	60.001	NA	(*7*)
18	A7536	Australia	2017	Asia-Pacific	0.5	1903	60.001	SRR6238584	(*8*)
19	A7846	Australia	2017	Asia-Pacific	0.5	1903	60.001	SRR6238583	(*8*)
20	47707	Canada	2017	Asia-Pacific	1	1903	60.001	SRR6191747	(*9*)
21	GC185	China	2017	Asia-Pacific	1	1903	60.001	SRR10403302	(*10*)
22	GC195	China	2017	Asia-Pacific	1	1903	60.001	SRR10403301	(*10*)
23	GC196	China	2017	Asia-Pacific	1	1903	60.001	SRR10403300	(*10*)
24	DG17067	China	2017	Asia-Pacific	0.5	7365	60.001	SRR21311932	(*11*)
25	GK124	Denmark	2017	No	0.5	1903	60.001	GCA_900205725.1	(*12*)
26	KM383	Japan	2017	Asia-Pacific	0.5	1903	60.001	DRR099845	(*1*)
27	KU17039	Japan	2017	Asia-Pacific	0.5	1903	60.001	DRR146960	(*1*)
28	NJ1711654	China	2017	Asia-Pacific	1	1903	60.001	GCA_007107165.1	(*13*)
29	BJ18014	China	2018	Asia-Pacific	0.5	1600	60.001	NA	(*4*)
30	BJ18035	China	2018	Asia-Pacific	1	1600	60.001	NA	(*4*)
31	MM14	China	2018	Asia-Pacific	0.5	1903	60.001	SRR16847905	(*5*)
32	ZH545	China	2018	Asia-Pacific	0.5	7365	60.001	SRR16847907	(*5*)
33	A2543	Australia	2018	Asia-Pacific	0.5	12039	60.001	ERR2865779	(*14*)
34	A2735	Australia	2018	Asia-Pacific	0.25	12039	60.001	ERR2865780	(*14*)
35	51742	Canada	2018	Asia-Pacific	0.5	1903	60.001	SRR8695957	(*15*)
36	GC249	China	2018	Asia-Pacific	0.5	7365	60.001	SRR10403299	(*10*)
37	GC250	China	2018	Asia-Pacific	0.5	7365	60.001	SRR10403298	(*10*)

Number	Isolate	Country	Year	Local, travel or contact region	CRO (mg/L)	MLST	*pen*A allele	Genomic accession no.	References
38	SC18–25	China	2018	Asia-Pacific	≥0.5	1903	60.001	SRR10289965	(*16*)
39	SC18–26	China	2018	Asia-Pacific	≥0.5	1903	60.001	SRR10289964	(*16*)
40	SC18–33	China	2018	Asia-Pacific	≥0.5	1903	60.001	SRR10289963	(*16*)
41	SC18–68	China	2018	Asia-Pacific	≥0.5	7363	60.001	SRR10289962	(*16*)
42	DG18193	China	2018	Asia-Pacific	0.5	1903	60.001	SRR16847903	(*5*)
43	GD69	China	2018	Asia-Pacific	0.5	1903	60.001	SRR21311933	(*11*)
44	IR72	Ireland	2018	Asia-Pacific	0.5	1903	60.001	GCA_900624675.1	(*17*)
45	18DG342	Singapore	2018	Asia-Pacific	1	13871	60.001	SRR8369788	(*18*)
46	G97687	United Kingdom	2018	Asia-Pacific	0.5	12039	60.001	ERR2560140	(*19*)
47	G7944	United Kingdom	2018	No	0.5	12039	60.001	ERR2560139	(*19*)
48	H18–209	United Kingdom	2018	Asia-Pacific	1	1903	60.001	SRR8695248	(*20*)
49	H18–502	United Kingdom	2018	Asia-Pacific	1	1903	60.001	SRR8695247	(*20*)
50	NJ189125	China	2018	Asia-Pacific	1	1903	60.001	GCA_007107365.1	(*13*)
51	SS74	China	2019	Asia-Pacific	0.5	1903	60.001	SRR16847906	(*5*)
52	GZ19–42	China	2019	Asia-Pacific	0.5	1903	60.001	NA	This study
53	ZJ19-F1	China	2019	Asia-Pacific	0.25	1600	60.001	NA	This study
54	SRRSH203	China	2019	Asia-Pacific	1	1600	60.001	GCA_016802445.1	(*21*)
55	SRRSH204	China	2019	Asia-Pacific	1	1600	60.001	GCA_016802425.1	(*21*)
56	SRRSH205	China	2019	Asia-Pacific	1	1600	60.001	GCA_016802405.1	(*21*)
57	SRRSH207	China	2019	Asia-Pacific	1	1903	60.001	GCA_016802385.1	(*21*)
58	SRRSH214	China	2019	Asia-Pacific	1	1600	60.001	GCA_016802355.1	(*21*)
59	SRRSH229	China	2019	Asia-Pacific	1	1600	60.001	GCA_016802335.1	(*21*)
60	SRRSH240	China	2019	Asia-Pacific	1	1903	60.001	GCA_016802315.1	(*21*)
61	DG19112	China	2019	Asia-Pacific	0.5	13943	60.001	SRR16847904	(*5*)
62	VN226T	Vietnam	2019	Asia-Pacific	0.5	13871	60.001	ERR6139617	(*22*)
63	VN233T	Vietnam	2019	Asia-Pacific	0.38	13871	60.001	ERR6139616	(*22*)

Number	Isolate	Country	Year	Local, travel or contact region	CRO (mg/L)	MLST	*pen*A allele	Genomic accession no.	References
64	VN256T	Vietnam	2019	Asia-Pacific	0.06	13871	60.001	ERR6139602	(*22*)
65	NJ195417	China	2019	Asia-Pacific	1	1903	60.001	GCA_030645065.1	(*13*)
66	NJ196610	China	2019	Asia-Pacific	1	1903	60.001	GCA_030645045.1	(*13*)
67	NJ197542	China	2019	Asia-Pacific	1	1903	60.001	GCA_030645025.1	(*13*)
68	NJ1911400	China	2019	Asia-Pacific	1	1903	60.001	GCA_030645005.1	(*13*)
69	NJ1913940	China	2019	Asia-Pacific	0.5	1903	60.001	GCA_030644985.1	(*13*)
70	NJ1914215	China	2019	Asia-Pacific	1	7363	60.001	GCA_030644965.1	(*13*)
71	NJ1914646	China	2019	Asia-Pacific	0.5	1903	60.001	GCA_030644945.1	(*13*)
72	GCWGS-10753	America	2019	Asia-Pacific	1	1901	60.001	SRR16999585	(*23*)
73	NG19051	China	2019	Asia-Pacific	>2	1901	60.001	SRR19962642	(*24*)
74	CD19–21	China	2019	Asia-Pacific	0.5	1903	60.001	SRR23314763	This study
75	CD19–46	China	2019	Asia-Pacific	0.5	1903	60.001	SRR23314762	This study
76	CD19–81	China	2019	Asia-Pacific	0.5	8123	60.001	SRR23314759	This study
77	CD19–97	China	2019	Asia-Pacific	0.5	1903	60.001	SRR23314758	This study
78	SH19–34	China	2019	Asia-Pacific	0.5	1903	60.001	SRR23342062	This study
79	SH19–72	China	2019	Asia-Pacific	0.5	1600	60.001	SRR23342061	This study
80	SH19–79	China	2019	Asia-Pacific	0.5	1903	60.001	SRR23342060	This study
81	SH19–101	China	2019	Asia-Pacific	0.5	1903	60.001	SRR23342059	This study
82	ZJ19-F9	China	2019	Asia-Pacific	0.25	1903	60.001	SRR23462773	This study
83	F91	France	2019	No	0.5	13871	60.001	GCA_007655285.1	(*25*)
84	VN04D	Vietnam	2020	Asia-Pacific	0.38	13871	60.001	NA	(*22*)
85	1669	China	2020	Asia-Pacific	0.25	1903	60.001	NA	(*26*)
86	NJ203279	China	2020	Asia-Pacific	1	7363	60.001	NA	(*13*)
87	GZ20–07	China	2020	Asia-Pacific	1	1600	60.001	NA	This study
88	GZ20–51	China	2020	Asia-Pacific	0.5	1903	60.001	NA	This study
89	ZJ20–231	China	2020	Asia-Pacific	1	1600	60.001	NA	This study

Number	Isolate	Country	Year	Local, travel or contact region	CRO (mg/L)	MLST	*pen*A allele	Genomic accession no.	References
90	GZ20–91	China	2020	Asia-Pacific	1	1903	60.001	SRR23325685	This study
91	YL201	China	2020	Asia-Pacific	0.75	1600	60.001	SRR15204538	(*27*)
92	VN75T	Vietnam	2020	Asia-Pacific	0.25	13871	60.001	ERR6139609	(*22*)
93	VN76T	Vietnam	2020	Asia-Pacific	0.5	13871	60.001	ERR6139610	(*22*)
94	VN43T	Vietnam	2020	Asia-Pacific	0.75	13871	60.001	ERR6139607	(*22*)
95	VN03D	Vietnam	2020	Asia-Pacific	0.25	13871	60.001	ERR6139613	(*22*)
96	VN34D	Vietnam	2020	Asia-Pacific	0.38	13871	60.001	ERR6139615	(*22*)
97	VN20T	Vietnam	2020	Asia-Pacific	0.75	13871	60.001	ERR6139605	(*22*)
98	VN37T	Vietnam	2020	Asia-Pacific	0.38	13871	60.001	ERR6139606	(*22*)
99	VN98T	Vietnam	2020	Asia-Pacific	0.38	13871	60.001	ERR6139611	(*22*)
100	VN128T	Vietnam	2020	Asia-Pacific	0.5	13871	60.001	ERR6139612	(*22*)
101	VN44T	Vietnam	2020	Asia-Pacific	0.06	13871	60.001	ERR6139608	(*22*)
102	NJ204705	China	2020	Asia-Pacific	1	11710	60.001	GCA_030644925.1	(*13*)
103	NJ208430	China	2020	Asia-Pacific	0.5	1903	60.001	GCA_030644905.1	(*13*)
104	NJ208756	China	2020	Asia-Pacific	1	1903	60.001	GCA_030644885.1	(*13*)
105	NJ209649	China	2020	Asia-Pacific	1	7363	60.001	GCA_030644865.1	(*13*)
106	NG20092	China	2020	Asia-Pacific	0.5	1903	60.001	SRR19962359	(*24*)
107	NG20320	China	2020	Asia-Pacific	>0.5	1901	60.001	SRR19962491	(*24*)
108	NG20402	China	2020	Asia-Pacific	>0.5	1903	60.001	SRR19962431	(*24*)
109	NG20403	China	2020	Asia-Pacific	>0.5	1903	60.001	SRR19962430	(*24*)
110	NG20404	China	2020	Asia-Pacific	>0.5	1903	60.001	SRR19962429	(*24*)
111	NG20405	China	2020	Asia-Pacific	>0.5	1903	60.001	SRR19962428	(*24*)
112	NG20268	China	2020	Asia-Pacific	>0.5	1903	60.001	SRR19962516	(*24*)
113	NG20105	China	2020	Asia-Pacific	>0.5	1903	60.001	SRR19962159	(*24*)
114	NG20139	China	2020	Asia-Pacific	>0.5	1903	60.001	SRR19962596	(*24*)
115	NG20267	China	2020	Asia-Pacific	0.5	1903	60.001	SRR19962517	(*24*)

Number	Isolate	Country	Year	Local, travel or contact region	CRO (mg/L)	MLST	*pen*A allele	Genomic accession no.	References
116	NG20276	China	2020	Asia-Pacific	>0.5	1903	60.001	SRR19962731	(*24*)
117	NG20283	China	2020	Asia-Pacific	>0.5	1903	60.001	SRR19962723	(*24*)
118	NG20285	China	2020	Asia-Pacific	0.5	1903	60.001	SRR19962721	(*24*)
119	NG20385	China	2020	Asia-Pacific	>0.5	7365	60.001	SRR19962450	(*24*)
120	CD20–6	China	2020	Asia-Pacific	0.5	1903	60.001	SRR23314754	This study
121	CD20–7	China	2020	Asia-Pacific	0.5	7356	60.001	SRR23314760	This study
122	CD20–37	China	2020	Asia-Pacific	0.25	8123	60.001	SRR23314756	This study
123	CD20–48	China	2020	Asia-Pacific	0.5	1903	60.001	SRR23314755	This study
124	CD20–59	China	2020	Asia-Pacific	0.0156	7822	60.001	NA	This study
125	CD20–60	China	2020	Asia-Pacific	0.5	1903	60.001	SRR23314753	This study
126	CD20–64	China	2020	Asia-Pacific	0.5	8123	60.001	SRR23314761	This study
127	SH20–95	China	2020	Asia-Pacific	0.5	13943	60.001	SRR23342058	This study
128	SH20–167	China	2020	Asia-Pacific	0.5	1903	60.001	SRR23342057	This study
129	ZJ20–23	China	2020	Asia-Pacific	0.5	1903	60.001	SRR23462772	This study
130	ZJ20–230	China	2020	Asia-Pacific	1	1600	60.001	SRR23462770	This study
131	ZJ20–215	China	2020	Asia-Pacific	1	1903	60.001	SRR23462771	This study
132	2021-A1	China	2021	Asia-Pacific	0.5	7827	60.001	NA	(*28*)
133	2021-A2	China	2021	Asia-Pacific	0.5	10314	60.001	NA	(*28*)
134	2021-A3	China	2021	Asia-Pacific	0.25	7365	60.001	NA	(*28*)
135	2021-A4	China	2021	Asia-Pacific	0.25	7365	60.001	NA	(*28*)
136	2021-A5	China	2021	Asia-Pacific	0.5	1903	60.001	NA	(*28*)
137	2021-A6	China	2021	Asia-Pacific	0.25	7365	60.001	NA	(*28*)
138	2021-A7	China	2021	Asia-Pacific	0.25	7365	60.001	NA	(*28*)
139	2021-A8	China	2021	Asia-Pacific	0.25	7365	60.001	NA	(*28*)
140	2021-A9	China	2021	Asia-Pacific	≥1	13871	60.001	NA	(*28*)
141	2021-A10	China	2021	Asia-Pacific	0.5	1903	60.001	NA	(*28*)

Number	Isolate	Country	Year	Local, travel or contact region	CRO (mg/L)	MLST	*pen*A allele	Genomic accession no.	References
142	2021-A11	China	2021	Asia-Pacific	0.25	1903	60.001	NA	(*28*)
143	2021-A12	China	2021	Asia-Pacific	0.5	1600	60.001	NA	(*28*)
144	CD21–4	China	2021	Asia-Pacific	0.125	8123	60.001	NA	This study
145	CD21–17	China	2021	Asia-Pacific	0.25	1903	60.001	NA	This study
146	CD21–27	China	2021	Asia-Pacific	0.25	7365	60.001	NA	This study
147	GZ21–10	China	2021	Asia-Pacific	0.016	1600	60.001	NA	This study
148	SH21–6	China	2021	Asia-Pacific	NA	1903	60.001	NA	This study
149	SH21–69	China	2021	Asia-Pacific	0.03	1903	60.001	NA	This study
150	SH21–76	China	2021	Asia-Pacific	NA	13943	60.001	NA	This study
151	SH21–145	China	2021	Asia-Pacific	0.03	1903	60.001	NA	This study
152	SH21–186	China	2021	Asia-Pacific	0.5	1901	60.001	NA	This study
153	SH21–197	China	2021	Asia-Pacific	NA	1903	60.001	NA	This study
154	SH21–232	China	2021	Asia-Pacific	0.5	7365	60.001	NA	This study
155	SH21–295	China	2021	Asia-Pacific	0.5	1903	60.001	NA	This study
156	SH21–296	China	2021	Asia-Pacific	NA	7371	60.001	NA	This study
157	SH21–358	China	2021	Asia-Pacific	0.25	1903	60.001	NA	This study
158	SH21–366	China	2021	Asia-Pacific	0.5	1903	60.001	NA	This study
159	ZJ21-test7	China	2021	Asia-Pacific	1	7363	60.001	NA	This study
160	ZJ21-test12	China	2021	Asia-Pacific	1	7363	60.001	NA	This study
161	GD2021236	China	2021	Asia-Pacific	0.5	1588	60.001	SRR24303598	(*29*)
162	GD2021027	China	2021	Asia-Pacific	0.5	10314	60.001	SRR24303599	(*29*)
163	GD2021273	China	2021	Asia-Pacific	0.5	1903	60.001	SRR24303597	(*29*)
164	GD2021291	China	2021	Asia-Pacific	0.5	7365	60.001	SRR24303596	(*29*)
165	GD2021270	China	2021	Asia-Pacific	0.5	1903	60.001	SRR24303590	(*29*)
166	GD2021272	China	2021	Asia-Pacific	0.5	1903	60.001	SRR24303588	(*29*)
167	GD2021265	China	2021	Asia-Pacific	0.5	7365	60.001	SRR24303595	(*29*)

Number	Isolate	Country	Year	Local, travel or contact region	CRO (mg/L)	MLST	*pen*A allele	Genomic accession no.	References
168	GD2021267	China	2021	Asia-Pacific	0.5	1903	60.001	SRR24303593	(*29*)
169	GD2021269	China	2021	Asia-Pacific	0.5	1903	60.001	SRR24303591	(*29*)
170	GD2021271	China	2021	Asia-Pacific	≥1	1903	60.001	SRR24303589	(*29*)
171	GD2021266	China	2021	Asia-Pacific	0.5	13943	60.001	SRR24303594	(*29*)
172	GD2021268	China	2021	Asia-Pacific	0.5	1903	60.001	SRR24303592	(*29*)
173	Case1	United Kingdom	2022	Asia-Pacific	0.25	8123	60.001	ERR10659307	(*30*)
174	Case2	United Kingdom	2022	Asia-Pacific	1	8123	60.001	ERR10659308	(*30*)
175	Case3	United Kingdom	2022	Asia-Pacific	1	8123	60.001	ERR10659309	(*30*)
176	Case4	United Kingdom	2022	Asia-Pacific	0.5	8123	60.001	ERR10659310	(*30*)
177	Case5	United Kingdom	2022	Asia-Pacific	0.5	8123	60.001	ERR10659311	(*30*)
178	Case6	United Kingdom	2022	Asia-Pacific	0.5	8123	60.001	ERR10659312	(*30*)
179	Case7	United Kingdom	2022	Asia-Pacific	0.25	8123	60.001	ERR10659313	(*30*)
180	Case8	United Kingdom	2022	Asia-Pacific	0.5	8123	60.001	ERR10659314	(*30*)
181	Case10	United Kingdom	2022	Asia-Pacific	0.25	16406	60.001	ERR10659316	(*30*)
182	AT159	Austria	2022	Asia-Pacific	0.5	16406	60.001	ERR9769205	(*31*)
183	SE690	Sweden	2022	No	0.25	8130	60.001	GCA_028483175.2	(*32*)
184	F93	France	2022	No	0.25	16406	60.001	GCA_032460605.1	(*33*)
185	F94	France	2022	Asia-Pacific	0.25	16406	60.001	GCA_032460585.1	(*33*)
186	SRR22570615	Cambodia	2022	Asia-Pacific	0.125	17961	60.001	SRR22570615	(*34*)
187	SRR22570614	Cambodia	2022	Asia-Pacific	0.25	1587	60.001	SRR22570614	(*34*)
188	SRR22570603	Cambodia	2022	Asia-Pacific	0.25	13871	60.001	SRR22570603	(*34*)
189	SRR22570594	Cambodia	2022	Asia-Pacific	0.25	1587	60.001	SRR22570594	(*34*)
190	SRR22570593	Cambodia	2022	Asia-Pacific	0.5	13871	60.001	SRR22570593	(*34*)
191	SRR22570592	Cambodia	2022	Asia-Pacific	0.125	8130	60.001	SRR22570592	(*34*)
192	SRR22570591	Cambodia	2022	Asia-Pacific	0.125	8130	60.001	SRR22570591	(*34*)
193	SRR22570590	Cambodia	2022	Asia-Pacific	0.5	13871	60.001	SRR22570590	(*34*)

Number	Isolate	Country	Year	Local, travel or contact region	CRO (mg/L)	MLST	*pen*A allele	Genomic accession no.	References
194	SRR22570589	Cambodia	2022	Asia-Pacific	0.5	13871	60.001	SRR22570589	(*34*)
195	SRR22570588	Cambodia	2022	Asia-Pacific	0.25	8130	60.001	SRR22570588	(*34*)
196	SRR22570613	Cambodia	2022	Asia-Pacific	0.25	8130	60.001	SRR22570613	(*34*)
197	SRR22570612	Cambodia	2022	Asia-Pacific	0.25	16406	60.001	SRR22570612	(*34*)
198	SRR22570611	Cambodia	2022	Asia-Pacific	0.125	7363	60.001	SRR22570611	(*34*)
199	SRR22570610	Cambodia	2022	Asia-Pacific	0.25	7363	60.001	SRR22570610	(*34*)
200	SRR22570609	Cambodia	2022	Asia-Pacific	0.125	8130	60.001	SRR22570609	(*34*)
201	SRR22570608	Cambodia	2022	Asia-Pacific	0.25	8130	60.001	SRR22570608	(*34*)
202	SRR22570607	Cambodia	2022	Asia-Pacific	0.125	8130	60.001	SRR22570607	(*34*)
203	SRR22570606	Cambodia	2022	Asia-Pacific	0.125	8130	60.001	SRR22570606	(*34*)
204	SRR22570605	Cambodia	2022	Asia-Pacific	0.25	1925	60.001	SRR22570605	(*34*)
205	SRR22570604	Cambodia	2022	Asia-Pacific	0.25	7363	60.001	SRR22570604	(*34*)
206	SRR22570602	Cambodia	2022	Asia-Pacific	0.25	7363	60.001	SRR22570602	(*34*)
207	SRR22570601	Cambodia	2022	Asia-Pacific	0.25	8143	60.001	SRR22570601	(*34*)
208	SRR22570600	Cambodia	2022	Asia-Pacific	0.25	13871	60.001	SRR22570600	(*34*)
209	SRR22570599	Cambodia	2022	Asia-Pacific	0.25	11368	60.001	SRR22570599	(*34*)
210	SRR22570598	Cambodia	2022	Asia-Pacific	0.25	7363	60.001	SRR22570598	(*34*)
211	SRR22570597	Cambodia	2022	Asia-Pacific	0.125	8130	60.001	SRR22570597	(*34*)
212	SRR22570596	Cambodia	2022	Asia-Pacific	0.5	16406	60.001	SRR22570596	(*34*)
213	SRR22570595	Cambodia	2022	Asia-Pacific	0.25	16406	60.001	SRR22570595	(*34*)
214	SRR24964567	Cambodia	2022	Asia-Pacific	0.5	13871	60.001	SRR24964567	(*34*)

CRO, ceftriaxone; MLST: multilocus sequence typing; NA, not available.

References

1. Lee K, Nakayama SI, Osawa K, Yoshida H, Arakawa S, Furubayashi KI, et al. Clonal expansion and spread of the ceftriaxone-resistant *Neisseria gonorrhoeae* strain FC428, identified in Japan in 2015, and closely related isolates. J Antimicrob Chemother. 2019;74:1812–9. PubMed https://doi.org/10.1093/jac/dkz129

2. Chen SC, Han Y, Yuan LF, Zhu XY, Yin YP. Identification of internationally disseminated ceftriaxone-resistant *Neisseria gonorrhoeae* strain FC428, China. Emerg Infect Dis. 2019;25:1427–9. PubMed https://doi.org/10.3201/eid2507.190172

3. Yang F, Zhang H, Chen Y, Zhai Y, Zhao F, Yu Y, et al. Detection and analysis of two cases of the internationally spreading ceftriaxone-resistant *Neisseria gonorrhoeae* FC428 clone in China. J Antimicrob Chemother. 2019;74:3635–6. PubMed https://doi.org/10.1093/jac/dkz384

4. Chen SC, Yuan LF, Zhu XY, van der Veen S, Yin YP. Sustained transmission of the ceftriaxone-resistant *Neisseria gonorrhoeae* FC428 clone in China. J Antimicrob Chemother. 2020;75:2499–502. PubMed https://doi.org/10.1093/jac/dkaa196

5. Lin X, Chen W, Xie Q, Yu Y, Liao Y, Feng Z, et al. Dissemination and genome analysis of high-level ceftriaxone-resistant *penA* 60.001 *Neisseria gonorrhoeae* strains from the Guangdong Gonococcal antibiotics susceptibility Programme (GD-GASP), 2016-2019. Emerg Microbes Infect. 2022;11:344–50. PubMed https://doi.org/10.1080/22221751.2021.2011618

6. Yang Y, Yang Y, Martin I, Dong Y, Diao N, Wang Y, et al. NG-STAR genotypes are associated with MDR in *Neisseria gonorrhoeae* isolates collected in 2017 in Shanghai. J Antimicrob Chemother. 2020;75:566–70. PubMed https://doi.org/10.1093/jac/dkz471

7. Poncin T, Fouere S, Braille A, Camelena F, Agsous M, Bebear C, et al. Multidrug-resistant *Neisseria gonorrhoeae* failing treatment with ceftriaxone and doxycycline in France, November 2017. Euro surveillance: European communicable disease bulletin. 2018 May;23(21).

8. Lahra MM, Martin I, Demczuk W, Jennison AV, Lee KI, Nakayama SI, et al. Cooperative recognition of internationally disseminated ceftriaxone-resistant *Neisseria gonorrhoeae* strain. Emerg Infect Dis. 2018;24:735–40. PubMed https://doi.org/10.3201/eid2404.171873

9. Lefebvre B, Martin I, Demczuk W, Deshaies L, Michaud S, Labbé AC, et al. Ceftriaxone-resistant *Neisseria gonorrhoeae*, Canada, 2017. Emerg Infect Dis. 2018;24:381–3. PubMed https://doi.org/10.3201/eid2402.171756

10. Yuan Q, Li Y, Xiu L, Zhang C, Fu Y, Jiang C, et al. Identification of multidrug-resistant *Neisseria gonorrhoeae* isolates with combined resistance to both ceftriaxone and azithromycin, China, 2017-2018. Emerg Microbes Infect. 2019;8:1546–9. PubMed https://doi.org/10.1080/22221751.2019.1681242

11. Lin X, Chen W, Yu Y, Lan Y, Xie Q, Liao Y, et al. Emergence and genomic characterization of *Neisseria gonorrhoeae* isolates with high levels of ceftriaxone and azithromycin resistance in Guangdong, China, from 2016 to 2019. Microbiol Spectr. 2022;10:e0157022. PubMed https://doi.org/10.1128/spectrum.01570-22

12. Terkelsen D, Tolstrup J, Johnsen CH, Lund O, Larsen HK, Worning P, et al. Multidrug-resistant *Neisseria gonorrhoeae* infection with ceftriaxone resistance and intermediate resistance to azithromycin, Denmark, 2017. Euro surveillance: European communicable disease bulletin. 2017 Oct;22(42).

13. Zhao Y, Le W, Genco CA, Rice PA, Su X. Increase in multidrug resistant *Neisseria gonorrhoeae* FC428-like isolates harboring the mosaic *penA* 60.001 gene, in Nanjing, China (2017-2020). Infect Drug Resist. 2023;16:4053–64. PubMed https://doi.org/10.2147/IDR.S408896

14. Whiley DM, Jennison A, Pearson J, Lahra MM. Genetic characterisation of *Neisseria gonorrhoeae* resistant to both ceftriaxone and azithromycin. Lancet Infect Dis. 2018;18:717–8. PubMed https://doi.org/10.1016/S1473-3099(18)30340-2

15. Berenger BM, Demczuk W, Gratrix J, Pabbaraju K, Smyczek P, Martin I. Genetic characterization and enhanced surveillance of ceftriaxone-resistant *Neisseria gonorrhoeae* strain, Alberta, Canada, 2018. Emerg Infect Dis. 2019;25:1660–7. PubMed https://doi.org/10.3201/eid2509.190407

16. Wang H, Wang Y, Yong G, Li X, Yu L, Ma S, et al. Emergence and genomic characterization of the ceftriaxone-resistant *Neisseria gonorrhoeae* FC428 clone in Chengdu, China. J Antimicrob Chemother. 2020;75:2495–8. PubMed https://doi.org/10.1093/jac/dkaa123

17. Golparian D, Rose L, Lynam A, Mohamed A, Bercot B, Ohnishi M, et al. Multidrug-resistant *Neisseria gonorrhoeae* isolate, belonging to the internationally spreading Japanese FC428 clone, with ceftriaxone resistance and intermediate resistance to azithromycin, Ireland, August 2018. Euro surveillance: European communicable disease bulletin. 2018 Nov;23(47).

18. Ko KKK, Chio MTW, Goh SS, Tan AL, Koh TH, Abdul Rahman NB. First case of ceftriaxone-resistant multidrug-resistant *Neisseria gonorrhoeae* in Singapore. Antimicrob Agents Chemother. 2019;63:e02624-18. PubMed https://doi.org/10.1128/AAC.02624-18

19. Jennison AV, Whiley D, Lahra MM, Graham RM, Cole MJ, Hughes G, et al. Genetic relatedness of ceftriaxone-resistant and high-level azithromycin resistant *Neisseria gonorrhoeae* cases, United Kingdom and Australia, February to April 2018. Euro surveillance: European communicable disease bulletin. 2019 Feb;24(8).

20. Eyre DW, Town K, Street T, Barker L, Sanderson N, Cole MJ, et al. Detection in the United Kingdom of the *Neisseria gonorrhoeae* FC428 clone, with ceftriaxone resistance and intermediate resistance to azithromycin, October to December 2018. Euro surveillance: European communicable disease bulletin. 2019 Mar;24(10).

21. Yan J, Chen Y, Yang F, Ling X, Jiang S, Zhao F, et al. High percentage of the ceftriaxone-resistant *Neisseria gonorrhoeae* FC428 clone among isolates from a single hospital in Hangzhou, China. J Antimicrob Chemother. 2021;76:936–9. PubMed https://doi.org/10.1093/jac/dkaa526

22. Trinh TM, Nguyen TT, Le TV, Nguyen TT, Ninh DT, Duong BH, et al. *Neisseria gonorrhoeae* FC428 subclone, Vietnam, 2019-2020. Emerg Infect Dis. 2022;28:432–5. PubMed https://doi.org/10.3201/eid2802.211788

23. Reimche JL, Clemons AA, Chivukula VL, Joseph SJ, Schmerer MW, Pham CD, et al.; Antimicrobial-Resistant Working Group. Genomic analysis of 1710 surveillance-based *Neisseria gonorrhoeae* isolates from the USA in 2019 identifies predominant strain types and chromosomal antimicrobial-resistance determinants. Microb Genom. 2023;9:mgen001006. PubMed https://doi.org/10.1099/mgen.0.001006

24. Wang D, Li Y, Zhang C, Zeng Y, Peng J, Wang F. Genomic epidemiology of *Neisseria gonorrhoeae* in Shenzhen, China, during 2019-2020: increased spread of ceftriaxone-resistant isolates brings insights for strengthening public health responses. Microbiol Spectr. 2023;11:e0172823. PubMed https://doi.org/10.1128/spectrum.01728-23

25. Poncin T, Merimeche M, Braille A, Mainardis M, Bebear C, Jacquier H, et al. Two cases of multidrug-resistant *Neisseria gonorrhoeae* related to travel in south-eastern Asia, France, June 2019. Euro surveillance: European communicable disease bulletin. 2019 Sep;24(36).

26. Lin X, Qin X, Wu X, Liao Y, Yu Y, Xie Q, et al. Markedly increasing antibiotic resistance and dual treatment of *Neisseria gonorrhoeae* isolates in Guangdong, China, from 2013 to 2020. Antimicrob Agents Chemother. 2022;66:e0229421. PubMed https://doi.org/10.1128/aac.02294-21

27. Zhang L, Zhang C, Zeng Y, Li Y, Huang S, Wang F, et al. Emergence and characterization of a ceftriaxone-resistant *Neisseria gonorrhoeae* FC428 clone evolving moderate-level resistance to azithromycin in Shenzhen, China. Infect Drug Resist. 2021;14:4271–6. PubMed https://doi.org/10.2147/IDR.S336212

28. Tang Y, Liu X, Chen W, Luo X, Zhuang P, Li R, et al. Antimicrobial resistance profiling and genome analysis of the *penA*-60.001 *Neisseria gonorrhoeae* clinical isolates in China in 2021. J Infect Dis. 2023;228:792–9. PubMed https://doi.org/10.1093/infdis/jiad258

29. Liao Y, Xie Q, Li X, Yin X, Wu X, Liu M, et al. Dissemination of *Neisseria gonorrhoeae* with decreased susceptibility to extended-spectrum cephalosporins in Southern China, 2021: a genome-wide surveillance from 20 cities. Ann Clin Microbiol Antimicrob. 2023;22:39. PubMed https://doi.org/10.1186/s12941-023-00587-x

30. Day M, Pitt R, Mody N, Saunders J, Rai R, Nori A, et al. Detection of 10 cases of ceftriaxone-resistant *Neisseria gonorrhoeae* in the United Kingdom, December 2021 to June 2022. Euro surveillance: European communicable disease bulletin. 2022 Nov;27(46).

31. Pleininger S, Indra A, Golparian D, Heger F, Schindler S, Jacobsson S, et al. Extensively drug-resistant (XDR) *Neisseria gonorrhoeae* causing possible gonorrhoea treatment failure with ceftriaxone plus azithromycin in Austria, April 2022. Euro surveillance: European communicable disease bulletin. 2022 Jun;27(24).

32. Golparian D, Vestberg N, Sodersten W, Jacobsson S, Ohnishi M, Fang H, et al. Multidrug-resistant *Neisseria gonorrhoeae* isolate SE690: mosaic penA-60.001 gene causing ceftriaxone resistance internationally has spread to the more antimicrobial-susceptible genomic lineage, Sweden, September 2022. Euro surveillance: European communicable disease bulletin. 2023 Mar;28(10).

33. Maubaret C, Camelena F, Mrimeche M, Braille A, Liberge M, Mainardis M, et al. Two cases of extensively drug-resistant (XDR) *Neisseria gonorrhoeae* infection combining ceftriaxone-resistance and high-level azithromycin resistance, France, November 2022 and May 2023. Euro surveillance: European communicable disease bulletin. 2023 Sep;28(37).

34. Ouk V, Pham CD, Wi T, van Hal SJ, Lahra MM, group ECw. The Enhanced Gonococcal Surveillance Programme, Cambodia. The Lancet Infectious diseases. 2023 Sep;23(9):e332-e3.

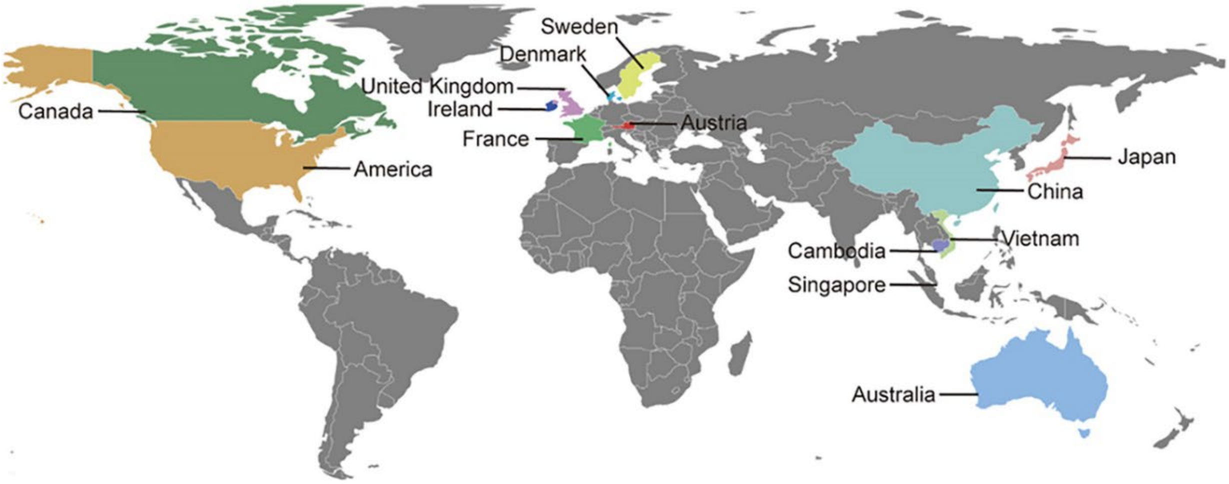

Appendix Figure. Data from a study of the surge in ceftriaxone-resistant *Neisseria gonorrhoeae* FC428-like strains in the Asia-Pacific region, 2015−2022. Distribution map showing global dissemination of ceftriaxone-resistant *Neisseria gonorrhoeae* FC428-like strains.
